# Is VATS approach suitable in re-operations for postoperative hemothorax after pulmonary resection? Data analysis in a big volume thoracic center

**DOI:** 10.1186/s13019-022-02099-9

**Published:** 2022-12-14

**Authors:** Zhixin Li, Lei-Lei Wu, Jiani Gao, Yichao Wang, Xiaogang Zhao, Dong Xie

**Affiliations:** Department of Thoracic Surgery, Shanghai Pulmonary Hospital, School of Medicine, Tongji University, No. 507 Zhengmin Road, Shanghai, 200433 People’s Republic of China

**Keywords:** Reoperation, Postoperative hemothorax, Pulmonary resection, Video-assisted thoracoscopic surgery

## Abstract

**Objective:**

This study explored the safety and of feasibility of video-assisted thoracoscopy (VATS) in re-operations for post-operative hemothorax.

**Methods:**

The clinical data of patients underwent re-operations due to post-operative hemothorax after pulmonary resection in Shanghai Pulmonary Hospital from 2006 to 2018 were retrospectively analysed. The incidence of re-operations were analyzed. The mortality and morbidity were compared between thoracotomy and thoracoscopic procedure for re-exploration.

**Results:**

A total of 114 patients were included. The annual incidence rate ranged from 0.21 to 0.54%; the perioperative mortality was 2.6%; there were 114 cases of re-operations for hemothorax after 2012, including 62 cases in thoracoscopy group and 52 cases in open group. The durations of chest-tube drainage (7.2 ± 3.9 days vs 10.9 ± 12.0 days, *P* = 0.001) and length of stay in hospital (13.7 ± 6.7 days vs 18.9 ± 10.6 days, *P* = 0.002) in the thoracoscopic group were shorter than those in the open group. The thoracoscopic group had fewer post-operative complications as well (*P* = 0.023). Meanwhile, post-operative complications in the delayed group were significantly higher than those in the non-delayed group, with a longer length of hospital stay and higher hospitalization costs.

**Conclusion:**

Complete VATS is safe and feasible for re-operation due to post-operative hemothorax and can be an alternative to thoracotomy. Delayed re-operations are associated with more post-operative complications and higher costs.

## Background

Thoracoscopic technology has been widely used in the surgical treatment of various thoracic diseases. Compared with traditional thoracotomy, increasing evidence has verified the advantges of performing thoracoscopic surgery: less post-operative pain, shorter length of hospital stay, fewer complications, an enhanced recovery and a better postoperative quality of life [1–3].

Post-operative hemothorax is one of the most critical complications in general thoracic surgery [4]. The typical progression of hemothorax occurs in three ways: complete spontaneous re-absorption of blood within several weeks, progression to fibrothorax, or infection with empyema formation [5]. In hemodynamically unstable patients with more than 1000 ml of blood drainage from the initial thoracotomy or ongoing blood loss of more than 100–200 ml/h lasting for 3 h, an intervention of surgical re-exploration with ongoing resuscitation is always imperatively required [6, 7]. Once the volume of hemothorax reaches 300 ml/h, timely and effective surgical hemostasis is critical. In earlier clinical practice, a thoracotomy approach was the first choice and preferred procedure for a surgical re-exploration and hemostasis. With the development of thoracoscopic techniques and early detection, more and more thoracoscopic surgery were perfomed for emergency condition (such as spontaneous pneumothorax, hemothorax or thoracic trauma) of thoracic patients. However, the benefits of the thoracoscopic procedure in these emergency thoracic surgery remain unclear. This study aims to evaluate the safety and feasibility of thoracoscopic exploration in post-operative hemothorax compared with an open approach after pulmonary resection.

## Materials and methods

### Patients

Patients from January 2006 to December 2018 in the Department of Thoracic Surgery of Shanghai Pulmonary Hospital were retrospectively analyzed. Inclusion criteria: (1)post-operative hemothorax occurred after primary pulmonary resection; (2)preoperative coagulation function was normal; (3)a re-operation was performed. Exclusion criteria: (1)incomplete clinical data; (2) thoracic re-operations for reasons other than post-operative hemothorax. Reoperation is defined as returning for surgical intervention due to post-operative excessive hemothorax from postanesthetic recovery room, the intensive care unit (ICU) or ward. The decision making of reoperation was made by the same consultant surgeon team based on the hemorrhage severity and condition of patients. A reoperation for fatal hemorrhage caused by great vessels rupture during operating room was excluded for our our comparative study. Because these catastrophic situations often ruled out a VATS attempt and immediate thoracotomy and bleeding control were often the first choice in our center. The indications for re-operation in our center were summarized as follows [4, 8, 9]: (1)hemodynamic instability (shock symptoms)caused by post-operative hemothorax; (2) continuous chest drainage ≥ 200 ml/h for more than 3 h or continuous chest drainage ≥ 100–150 ml/h for more than 5 h; (3)hemoglobin of the drainage was up to 60 g/L; (4) tension hemothorax due to post-operative hemothorax; (5)X-ray indicated massive hemotoma in chest cavity after a series of conservative treatment. In this study, causes of hemothorax were divided into 3 groups according to the sources of hemorrhage: arterial hemorrhage, venous hemorrhage and capillary hemorrhage [9] (See Classification in Table 1). This study was approved by the review board of our hospital, and the requirement for informed consent for the use of patients’ medical record was waived. All methods were performed in accordance with the Declaration of Helsinki.

**Table 1:** Classification and source of postoperative hemorrhage

Source of hemorrhage	Arterial hemorrhage	Venous hemorrhage	Capillary hemorrhage
	Bronchial arterial bleeding	Active bleeding of interlobar veins	Exudation of lung tissue surface
	Active artrial bleeding of incision	Active bleeding of intercostal veins	Exudation of chest wall
	Intercostal arterial bleeding	Active bleeding of superior vena cava	Diaphragmatic exudation
	Arterial bleeding of pleural adhesion	Active bleeding of stump of pulmonary vein	No obvious bleeding point but exudation
	Arterial bleeding of pulmonary ligament	Active bleeding of azygos vein/semiazygos vein	
	Active bleeding of pulmonary artery stump		
	Abnormal arterial bleeding around vagus nerve/phrenic nerve		
	Lymph node arterial bleeding		

### Surgical methods

All patients were in lateral decubitus position and underwent single-lung anesthesia with double-lumen endotracheal intubation. VATS procedure was first introduced for re-exploration for post-operative hemothorax in 2012 in our center. Generally, the incision length for re-operation was about 2–5 cm in the complete thoracoscopic group, which were performed under non-direct view without intercostal rib spreading. The thoracoscopic port location was determined by the primary surgical operation. A single incision (about 3 cm) was made through the 4/5th intercostal space in uni-portal VATS. While in multi-portal VATS, the observation port and operation port were selected in 7th and 4th intercostal space or 8th and 5th intercostal space respectively. In open thoracotomy reoperation group, a conventional posterolateral incision (about 25–30 cm) was made through the 4/5th intercostal space and operation was performed under direct vision and hand control. VATS-assisted procedure in our study was defined as video-assisted mini-thoracotomy, in which procedure surgical operation were carried out using a combination of direct vision and video-assistance. Cases underwent either complete VATS exploration or VATS-assisted reoperations were classified as VATS reoperation group in our study. Hemostasis methods were selected according to the surgical situation, including electrocoagulation, ligation of vessels, suture, and other physical or chemical methods. In our study, surgical reoperation beyond 48 h after the primary surgical procedure was defined as delayed exploration.

### Perioperative outcome

Post-operative complications were defined as all complications occured during or after hospitalization. All complications were evaluated and defined according to the Clavien-Dindo classification of surgical complications [10]. Complications requiring any drug or invasive intervention are defined as grade 2 or higher. Post-operative death is defined as any cause of death within 30 days after surgery.

### Statistical analysis

Data on patient's sex, age, Charlson comorbidity index (CCI), the durations of chest-tube drainage, post-operative hospital stay and major post-operative complications were collected. Demographic data and results were calculated using SPSS 23.0 software (SPSS Inc, Chicago, IL). T-test was used for continuous variables of normal distribution, while Mann–Whitney U-test was used for variables of non-normal distribution. The Pearson *x*^*2*^ test or the Fisher exact test, when appropriate, was used to compare proportions; a value of *P* < 0.05 was considered statistically significant.

## Results

**Fig. 1:**
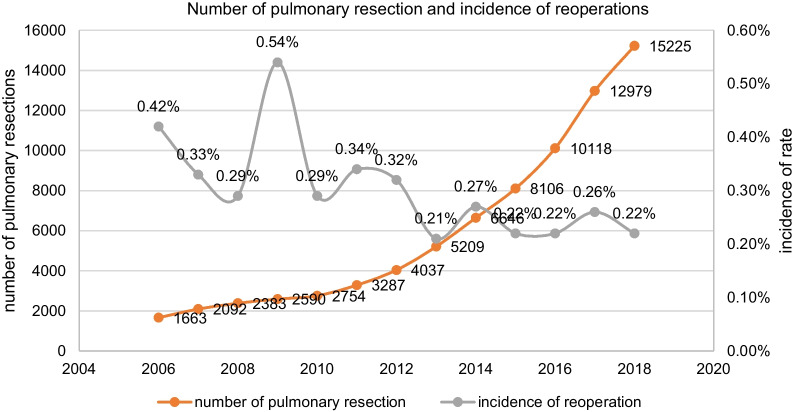
Incidence rate of re-operations for postoperative hemothroax after pulmonary resection per year in Shanghai Pulmonary Hospital from 2006 to 2018

From January 2006 to December 2018, a total of 203 re-operations for post-operative hemothorax after pulmonary resections were performed in the Department of Thoracic Surgery of Shanghai Pulmonary Hospital. The number of pulmonary resections performed per year and the incidence of re-operation were showed in Fig. 1. There was a rapid increase in the total number of pulmonary resections, but a relatively steady incidence rate for re-operation (ranging from 0.21 to 0.54%).

**Fig. 2:**
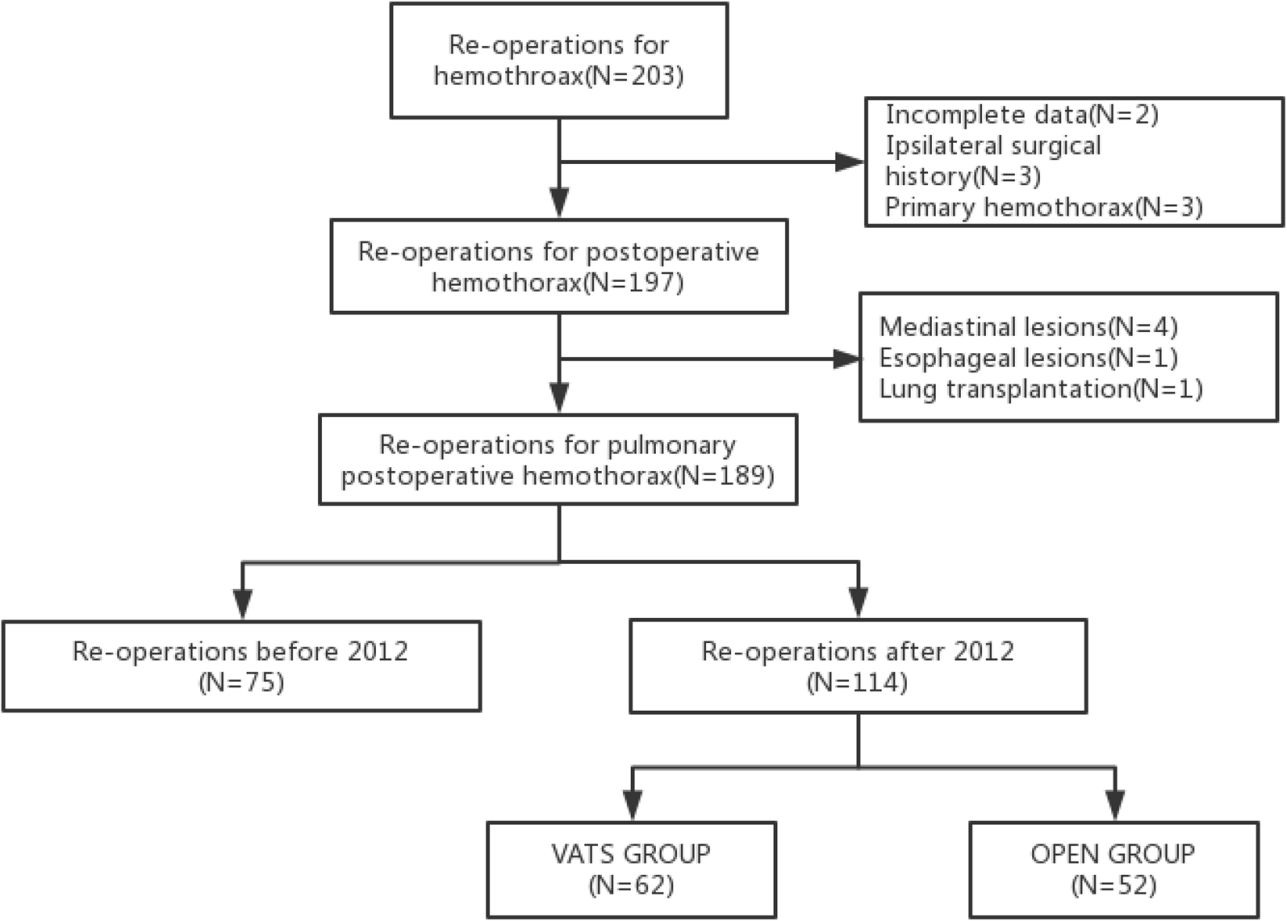
Flowchart of case selection

A total of 114 reoperations after primary pulmonary resections were included in our final study, including 87 patients with lung cancer and 27 patients with benign pulmonary lesion (including pulmonary bulla in 5 cases, aspergilloma in 3 cases, lung abscess and pneumonia in 2 cases, granulomatous inflammation in 5 cases, bronchial cyst with bronchiectasis in 7 cases, and other benign lesions in 5 cases) (Flow chart in Fig. 2). Among them, male patients accounted for 76.3%. The median age was 57.8 (16–85) years old. Patient characteristics were showed in Table 2. As for the cause of massive post-operative hemothorax, 74 (64.9%) cases were caused by arterial hemorrhage; 12 (10.5%) cases were caused by venous hemorrhage; 28 (24.6%) cases were caused by capillary hemorrhage; There were 39 arterial hemorrhage, 7 venous hemorrhage and 16 capillary hemorrhage in VATS reoperation group, while there were 35 arterial hemorrhage, 5 venous hemorrhage and 12 capillary hemorrhage in open reoperation group. There is of no significant difference in bleeding source between the two groups. The conversion rate of VATS for primary surgery in our study is 14.3% (10/70); Our study consisted of 50 complete VATS reoperations, 12 VATS assisted reoperations, and 52 direct open thoracotomy reoperations. We divided the procedure into the following groups, combining the primary surgical procedure and re-operation procedure: 54 patients in VATS-VATS group, 6 cases in VATS-open group, 5 cases in the VATS assisted-VATS group, and 5 cases in the VATS-assisted-open group, 44 cases in the open-VATS group. The VATS utilization rate in primary surgical procedure of VATS reoperations group is much higher than that in the primary surgical procedure of open reoperations group, which is of significant statistical difference (*P* = 0.001).

**Table 2:** Baseline and charateristics of patients who underwent reoperation for postoperative bleeding

	VATS group	Open group	*P* value
Gender			0.056
Male	43	44	
Female	19	8	
Age (y)	55.8 ± 14.2	60.1 ± 9.1	0.056
Charlson comorbidity index (CCI)			0.333
< 2	57	48	
≥ 2	5	4	
*Preoperative coagulation index*			
PT (s)	11.0 ± 0.8	11.0 ± 0.9	0.479
APTT (s)	32.1 ± 4.3	31.0 ± 5.2	0.213
Side			0.422
Left	30	19	
Right	32	33	
Lesion			0.396
Benign	14	13	
Malignant	48	39	
Total drainage before re-exploration (ml)	1233 (782–1500)	1365 (800–1637)	0.051
Primary procedure			0.001
VATS	54	6	
VATS-assisted	5	5	
Open	3	41	
The time from indication to re-operation (h)	24.0 (12.0–24.0)	24.0 (12.0–48.0)	0.450
Classification of bleeding source			0.884
Arterial hemorrhage	39	35	
Venous hemorrhage	7	5	
Capillary hemorrhage	16	12	

The mortality in our study was 2.6% (3/114), of which 2 died of respiratory failure due to ARDS, and 1 died of heart failure due to pulmonary embolism. There was 1 re-reoperations due to another post-operative hemothorax in the open group. The post-operative complication rate of grade 2 or above was 22.6% (13/62) in the VATS group, with a significant difference (*P* = 0.039) compared with the open-reoperation group (40.4%). In the meantime, complication rate was much lower in non-delayed group when compared to that in the delayed group (28.6% vs 43.8%, *P* = 0.047). The durations of chest-tube drainage in the thoracoscopic group was 6.0 (4.0–9.0) days, while it was 9.0 (6.0–11.7) days in the open group, with a statistically significant difference between the two groups (*P* < 0.001). Hospital stay in the thoracoscopic group was also significantly shorter than that in the open group (*P* = 0.002). Total hospitalization cost was nearly equivalent between the thoracoscopic group and the open group (*P* = 0.477). Although there was no significant difference in post-operative chest-tube drainage length between non-delayed group and delayed group (*P* = 0.230), non-delay group showed a shorter length of hospital stay (*P* = 0.026) and lower costs (*P* = 0.034) (Tables 3 and 4).

**Table 3:** Postoperative outcome and cost comparison between VATS group and open group

Variable	VATS group	Open group	*P* value
Complication			0.023
Grade 0–1	48	31	
Grade 2	9	13	
Grade 3–5	1	8	
Cost (US dollars)	10,016.9 (8793.1–12,454.6)	10,214.9 (8644.9–12,376.8)	0.522
Chest-tube drainage length (d)	6.0 (4.0–9.0)	9.0 (6.0–11.7)	0.001
Hospital stay (d)	12.0 (9.8–16.0)	16.5 (13.0–20.2)	0.002

**Table 4:** Postoperative outcome and cost comparison between non-delayed group and delayed group

Variable	Non-delayed group	Delayed group	*P* value
The time from indication to re-operation (h)	24.0 (12.0–24.0)	120.0 (96.0–210.0)	0.001
Complication			0.047
Grade 0–1	70	9	
Grade 2	22	3	
Grade 3–5	6	4	
Cost (US dollars)	9808.7 (8530.5–11,887.1)	12,287.8 (10,977–17,563.9)	0.034
Chest-tube drainage length (d)	7.0 (5.0–10.0)	8.0 (5.3–15.5)	0.230
Hospital stay (d)	13.0 (10.8–18.0)	20.0 (16.5–28.0)	0.026

## Discussion

Post-operative hemothorax is a critical complication in general thoracic surgery, which is characterized by rapid onset, strong concealment and heterogeneous causes. Timely surgical re-operation has always been an effective choice for postoperative hemothorax treatment after a failure of conservative therapeutic attempts. Descriptive reports of re-operations for postoperative complications are not rare [4, 8, 9, 11]. Sirbu et al. [11] summarized the causes of chest re-operations in 73 patients after pulmonary resections, of which post-operative hemothorax (38/72, 52%) accounted for nearly 50%. In Christophoros N’s [9] retrospective analysis of postoperative complications, the incidence of massive hemothorax had reached to approximately 1/3. Our study mainly focused on the role of VATS procedure in the management of postoperative emergency events, particularly in postoperative hemothorax. Our study indicated that reoperation procedures choices varied according to primary surgical procedures. The thoracoscopic procedure was preferred to be applied when the primary surgery was performed by completed thoracoscopic approach, while direct open thoracotomy procedure was more applied when primary surgery was performed in open thoracotomy approach. Due to a rapid advancement of minimally invasive technology and perioperative management experience, a significantly increasing trend in utilization of thoracoscopic procedures for re-operations was showed in our center.

### The effectiveness and feasibility of thoracoscopic reoperation

In recent years, minimally invasive procedure has developed rapidly and become as mainstream approach especially in thoracic surgery [12–14]. Improvements in equipment and technology have made it possible to perform minor and major pulmonary resections by complete VATS: such as lung biopsy, wedge resection, sleeve resection and even pneumonectomy. However, the application of VATS procedure in thoracic emergency events (massive thoracic bleeding or hemopneumothorax) is rarely reported. Solaini and Joshi et al. [15, 16] firstly reported that thoracoscopic techniques offered a better visual field and a better post-operative recovery in surgical treatment for traumatic hemopneumothorax. Our results had also confirmed a superior value of complete VATS approach in reoperation for post-operative massive hemorrhage. Under a similar severity of emergency situation, the thoracoscopic group obtained certain hemostasis effect without another massive bleeding after reoperations compared with open group. Our study has also indicated significantly less complications (22.6% vs 40.4%, *P* = 0.039) and shorter length of chest drainage and hospital stay in thoracoscopic reoperation group, which was consistent with the above literatures [17].

### The timing of re-operation

The decision for reoperation depends on effective post-operative monitoring and surgeons’ experience and the indications and timing of these reoperations varied greatly between different centers [18, 19]. Karthik et al. [20] and Choong et al. [21] respectively analyzed time-related risk factors of re-operation for massive post-operative hemothorax after cardiac surgery, suggesting that a delayed reoperation beyond 12 h might increase post-operative complications by nearly threefold (29% vs 7%, *P* < 0.05). In our study, the time from indication to re-operation in delayed group is significantly longer than that in non-delayed group. As a resut, the incidence rate of complications above Grade 2 in the delayed reoperation group was significantly higher than that in the non-delayed group (43.8% vs 28.6%, *P* = 0.047), which was consistent with the literature. Due to the fact that the majority of thoracic post-operative hemothorax were caused by arterial hemorrhage (64.9%, 74/114), delayed detection and postponed reoperation resulted in rapid blood loss and unstable hemodynamics, than unnecessary complications even after conservative treatments. In this respect, early detection of post-operative hemothorax and timely surgical intervention is critical for good recovery.

### Limitation

This is a retrospective study with inevitable selection bias. Heterogeneous hemorrhage causes and differentiated treatment for post-operative hemothorax made it complicated for data analysis. In addition, various patients’ conditions were also complex and heterogeneous, which could not be fully randomized to conduct a sufficient comparison. Moreover, severity of emergency situation could not be evaluated precisely without including data of blood coagulation and hemodynamic parameters during the reoperations.

## Conclusion

In conclusion, complete VATS is safe and feasible for reoperation due to post-operative hemothorax with a lower morbidity, shorter length of chest tube, shorter hospital stay compared to thoracotomy approach. Delayed re-operations are associated with more post-operative complications and higher costs. Under permissible conditions, timely thoracoscopic reoperation could be feasible and performed for post-operative hemothorax.

## Data Availability

The data that support the findings of this study are available from the corresponding author upon reasonable request.

## Abbreviations

VATS: Video-assisted thoracoscopic surgery
CCI: Charlson comorbidity index

## References

[1] Paul S, Altorki NK, Sheng S (2010). Thoracoscopic lobectomy is associated with lower morbidity than open lobectomy: a propensity-matched analysis from the STS database. J Thorac Cardiovasc Surg.

[2] Villamizar NR, Darrabie MD, Burfeind WR (2009). Thoracoscopic lobectomy is associated with lower morbidity compared with thoracotomy. J Thorac Cardiovasc Surg.

[3] Swanson SJ, Meyers BF, Gunnarsson CL (2012). Video-assisted thoracoscopic lobectomy is less costly and morbid than open lobectomy: a retrospective multiinstitutional database analysis. Ann Thorac Surg.

[4] Yang Y, Gao W, Zhao H (2016). Risk factors and consequences of perioperative reoperation in patients undergoing pulmonary resection surgery. Surgery.

[5] Carrillo EH, Richardson JD (1998). Thoracoscopy in the management of hemothorax and retained blood after trauma. Curr Opin Pulm Med.

[6] Casos SR, Richardson JD (2006). Role of thoracoscopy in acute management of chest injury. Curr Opin Crit Care.

[7] Kho SS, Yong MC, Chan SK (2018). Pulmonary arteriovenous malformation presenting as spontaneous haemothorax on transthoracic ultrasound. Thorax.

[8] van der Schaaf M, Derogar M, Johar A (2014). Reoperation after oesophageal cancer surgery in relation to long-term survival: a population-based cohort study. BMJ Open.

[9] Foroulis CN, Kleontas A, Karatzopoulos A (2014). Early reoperation performed for the management of complications in patients undergoing general thoracic surgical procedures. J Thorac Dis.

[10] Dindo D, Demartines N, Clavien PA (2004). Classification of surgical complications: a new proposal with evaluation in a cohort of 6336 patients and results of a survey. Ann Surg.

[11] Sirbu H, Busch T, Aleksic I (1999). Chest re-exploration for complications after lung surgery. Thorac Cardiovasc Surg.

[12] Cai J, Zhou J, Yang F, Wang J (2018). Adoption rate of video-assisted thoracic surgery for lung cancer varies widely in China. Chest.

[13] McElnay P, Casali G, Batchelor T, West D (2014). Adopting a standardized anterior approach significantly increases video-assisted thoracoscopic surgery lobectomy rates. Eur J Cardiothorac Surg.

[14] Divisi D, Bertolaccini L, Barone M (2018). National adoption of video-assisted thoracoscopic surgery (VATS) lobectomy: the Italian VATS register evaluation. J Thorac Dis.

[15] Solaini L, Prusciano F, Solaini L, Carletti M (2011). Video-assisted thoracoscopic surgery for postoperative hemothorax. Thorac Cardiovasc Surg.

[16] Joshi V, Kirmani B, Zacharias J (2013). Thoracotomy versus VATS: is there an optimal approach to treating pneumothorax?. Ann R Coll Surg Engl.

[17] Abd El-Hafez FM, Zahra A, Ghalwash M (2018). Thoracoscopy versus thoracotomy in hemodynamically stable patients with closed thoracic trauma. J Egypt Soc Cardio Thorac Surg.

[18] Ruel M, Chan V, Boodhwani M (2017). How detrimental is reexploration for bleeding after cardiac surgery?. J Thorac Cardiovasc Surg.

[19] Biancari F, Mikkola R, Heikkinen J (2012). Individual surgeon's impact on the risk of re-exploration for excessive bleeding after coronary artery bypass surgery. J Cardiothorac Vasc Anesth.

[20] Karthik S, Grayson AD, McCarron EE (2004). Reexploration for bleeding after coronary artery bypass surgery: risk factors, outcomes, and the effect of time delay. Ann Thorac Surg.

[21] Choong CK, Gerrard C, Goldsmith KA (2007). Delayed re-exploration for bleeding after coronary artery bypass surgery results in adverse outcomes. Eur J Cardiothorac Surg.

